# Maternal Provisioning of Offspring With Defence Chemicals in a Facultatively Parthenogenetic Stick Insect

**DOI:** 10.1002/ece3.71243

**Published:** 2025-04-24

**Authors:** Ana Caroline Oliveira Vasconcelos, Lewis Adler, Russell Bonduriansky

**Affiliations:** ^1^ School of Biological, Earth and Environmental Sciences University of New South Wales Sydney New South Wales Australia; ^2^ Bioanalytical Mass Spectrometry Facility, Mark Wainwright Analytical Centre University of New South Wales Sydney New South Wales Australia

**Keywords:** chemical defences, diet, maternal effect, *Megacrania batesii*, offspring provisioning

## Abstract

Parents can invest in offspring by transferring environmental factors, such as nutrients or diet‐derived defence chemicals, into eggs or embryos. However, in systems where females can reproduce facultatively without a male (facultative parthenogenesis), it is not known how reproductive mode and maternal environment affect offspring provisioning. The facultatively parthenogenetic stick insect *Megacrania batesii* sprays a defensive fluid from paired prothoracic glands. Here, we report that some hatchlings of *M. batesii* can spray even prior to their first feeding and provide evidence that both eggs and hatchlings contain the same diet‐derived chemical (the alkaloid actinidine) that is present in adult defensive spray. We also explored potential causes of variation among hatchlings in the capacity to spray, using a fully crossed experiment to investigate how offspring provisioning is affected by sexual versus parthenogenetic reproduction and high versus low maternal diet. We found that high maternal diet resulted in increased egg size but slower egg development, and maternal diet interacted with genotype to affect hatchling body size. Eggs laid by male‐paired females were larger, developed more quickly, and had higher hatching success by comparison with eggs laid by unpaired females, suggesting that mating and fertilisation enhance some aspects of offspring performance. However, hatchlings produced by unpaired females had larger prothoracic glands relative to body size than did hatchlings produced by male‐paired females, suggesting that sexual reproduction is associated with reduced provisioning of offspring with defensive chemicals. Our results reveal a novel example of maternal transfer of food‐derived defence chemicals to offspring and suggest that offspring provisioning with defence chemicals is affected by female reproductive mode.

## Graphical abstract

We show that females of the stick‐insect *Megacrania batesii* transfer defensive alkaloids to their offspring, providing a novel example of maternal provisioning. We also provide evidence that provisioning is enhanced by parthenogenetic reproduction in this species.

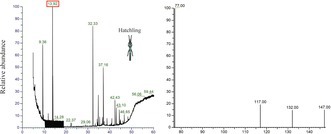

## 1 Introduction

Offspring can inherit a variety of components of the parental environment, such as nutrients, hormones, antibodies, and pathogens, that are transferred to offspring in the milk or yolk (Badyaev and Uller 2009; Bernardo 1996; Bonduriansky and Day 2018; Mousseau and Fox 1998; Uller 2008). However, the factors that are inherited from the parental environment can depend on parental condition (Bonduriansky 2021; Bonduriansky and Crean 2018; Qvarnström and Price 2001). Generally, nutrition plays a key role in influencing parental phenotype and condition, and consequently, the factors and resources that parents transfer to the next generation. For example, parents that acquire abundant food from their environment might be able to pass more resources to offspring (Bernardo 1996; Bonduriansky et al. 2016; Qvarnström and Price 2001). Offspring can also benefit if both mothers and fathers can invest resources in reproduction. For example, fathers can contribute resources in the seminal fluid, spermatophore, or nuptial gift, which might be incorporated by the female into the egg (e.g., Markow et al. 1990; Rapkin et al. 2016; Sternberg et al. 2015; Vahed 1998), or assist females in provisioning the offspring after hatching (Hunt and Simmons 1998, 2000).

An important component of the parental environment that offspring can inherit is factors associated with protection against predators or pathogens (Moran et al. 2010). When the environment that parents face induces defensive traits, the expression of those traits can also be induced in offspring through nongenetic factors as a form of transgenerational plasticity (Bell and Hellmann 2019; Bonduriansky 2021). For instance, plants that are damaged by herbivores can induce in the next generation higher expression of traits that offer protection against herbivory, such as defensive chemicals and trichomes (Agrawal et al. 1999). In some species, defensive compounds such as chemicals sequestered from the diet can be transmitted directly from parents to offspring. For example, in the strawberry poison frog *Oophaga pumilio* , mothers provision tadpoles with nutritive eggs that contain alkaloids (obtained by feeding on alkaloid‐containing arthropods) that can be consumed by the tadpoles and used as an antipredator defence. In this species, alkaloid titre in mothers is correlated with alkaloid titre in the tadpoles (Brooks et al. 2023), suggesting an important role of maternal diet in offspring provisioning. Similarly, mothers of the snake *Rhabdophis tigrinus* acquire defensive steroids from their food (toads) and transfer those compounds into the eggs, thereby provisioning hatchling snakes with antipredator chemical compounds (Hutchinson et al. 2008). Such provisioning can also occur in insects. For instance, larvae of the moth *Utetheisa ornatrix* sequester chemicals from their host–plant, carry those chemicals to the adult stage, and transfer them to the eggs, allowing individuals of all developmental stages to become unpalatable to predators (Eisner and Meinwald 1995). Likewise, the males of *U. ornatrix* can also supplement the chemicals that females invest into the eggs by transferring defence chemicals via the spermatophore (Eisner and Meinwald 1995).

The interactive nature of maternal and paternal effects and the mode of reproduction also have the potential to affect the transmission of environmental components to offspring (Champagne 2020; Verhoeven and Preite 2014). “Good genes” models of mate choice predict that female choosers might benefit by mating with males that carry high‐fitness alleles (Kirkpatrick 1996). Female preferences can also evolve if females obtain more direct benefits such as nutrients from certain males (Price et al. 1993). However, in systems where females can reproduce without a male (e.g., via facultative parthenogenesis), and possibly experience strong sexual conflict over mating (Burke and Bonduriansky 2017), it is unclear whether or how females might benefit by mating. For example, females of the facultatively parthenogenetic cockroach *Nauphoeta cinerea* that reproduced sexually had higher fitness and produced more offspring than females that reproduced parthenogenetically (Corley and Moore 1999). In the facultatively parthenogenetic stick insect *Extatosoma tiaratum*, sexually produced females had more offspring when mated than when reproducing parthenogenetically, whereas females that were produced parthenogenetically gained no fitness benefit by mating (Burke and Bonduriansky 2022). The net effects of sexual reproduction on female fitness in facultative parthenogens can therefore be complex, and the potential for sexual conflict certainly exists (see Section 4). However, sexual reproduction might enhance offspring fitness if sexual reproduction results in higher heterozygosity than parthenogenetic reproduction (Barton and Charlesworth 1998; D'Souza and Michiels 2010), or if males provide resources such as nutrients or defence chemicals in the seminal fluid (Crean et al. 2016; Eisner and Meinwald 1995; Vahed 1998). Males might also transfer substances (such as seminal fluid proteins from the male accessory glands) that stimulate females to lay eggs (Gillott 2003; Wigby et al. 2009; Wolfner 2002), or induce elevated investment in each offspring (Bonduriansky 2014). How maternal resources and reproductive mode affect investment in offspring in facultatively parthenogenetic animals is still poorly known.

**FIGURE 1:**
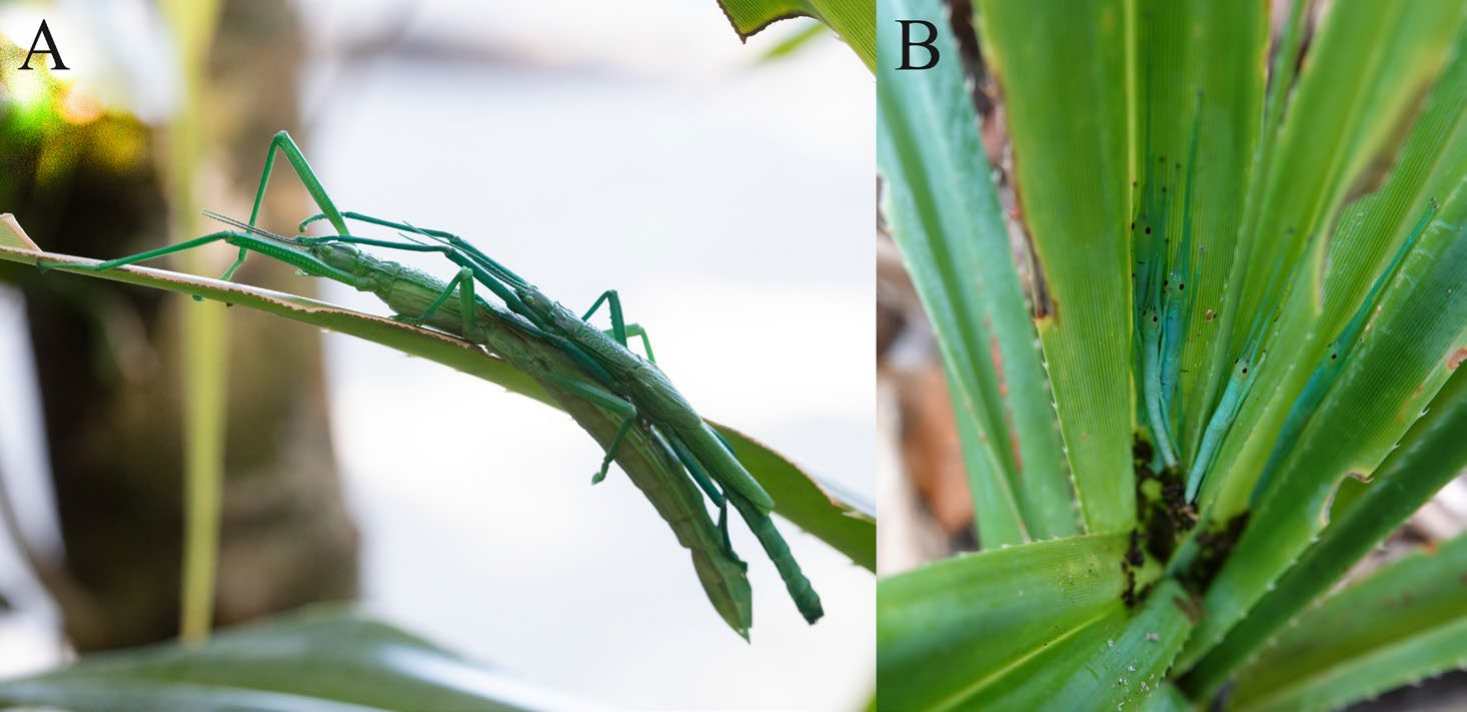
A *Megacrania batesii* female with a guarding male on her dorsum (A), and hatchling nymphs (B), on *Pandanus tectorius* host‐plants in Queensland, Australia. *M. batesii* females can mate and lay fertilised eggs that produce sons and daughters, or avoid mating and lay unfertilised eggs that develop parthenogenetically and produce daughters only. Populations vary in sex ratio and reproductive mode: Some populations exhibit near‐even sex ratios and mostly sexual reproduction, while other populations consist entirely of females and propagate exclusively via parthenogenesis.

The Peppermint Stick Insect, *Megacrania batesii*, is a facultatively parthenogenetic species that occurs in a spatial mosaic of all‐female populations (where reproduction is exclusively parthenogenetic) and mixed‐sex populations (where reproduction is primarily sexual) in the wet tropics of far‐north Queensland, Australia (Figure 1). Females from both all‐female and mixed‐sex populations of *M. batesii* are capable of sexual and parthenogenetic reproduction, producing all‐female broods from unfertilized eggs or mixed‐sex broods from fertilised eggs (Miller, Stuart, et al. 2024a; Wilner et al. 2025). Genomic data have shown that *M. batesii* populations are separated into two main genetic clusters (henceforth referred to as Southern and Northern genotypes), with the Southern genotype found mainly in all‐female populations and the Northern genotype found mainly in mixed‐sex populations (Miller, Stuart, et al. 2024a). Mixed‐sex and all‐female populations occur in similar habitat and use the same host‐plant species, such that reproductive mode appears to be determined solely by the presence of males and the extent of female resistance to mating and fertilisation (Miller, Wilner, et al. 2024b; Wilner et al. 2025). While the chemical composition of the defence fluid of *M. batesii* has not been investigated before, other species of *Megacrania* are known to sequester the chemical compound actinidine (or its chemical precursor) from their host‐plants (*Pandanus* sp. and *Benstonea* sp.) (Chow and Lin 1986; Ho and Chow 1993; Prescott et al. 2009). *Megacrania* accumulates this alkaloid in a pair of specialised glands located in the prothorax that function to store and spray the defence fluid (Chow and Lin 1986). In response to simulated predator attack, *Megacrania batesii* sprays its defence fluid directionally from the left and/or right prothoracic glands via movable ducts that protrude from the anterior corners of the prothorax (Jones and Bulbert 2020; Video 1). Actinidine also has antifungal properties that could prevent infections from fungal pathogens (Saxena and Mathela 1996). The defence fluid of *Megacrania* has a distinctive peppermint‐like odour from which the common name of this insect derives.

**VIDEO 1:** *Megacrania batesii* adult female spraying defence fluid. The female, perched on a *Benstonea monticola* host‐plant, was gently prodded with a stick to elicit a defensive reaction. The video was recorded at 30 frames per second. Video content can be viewed at https://onlinelibrary.wiley.com/doi/10.1002/ece3.71243

**FIGURE 2:**
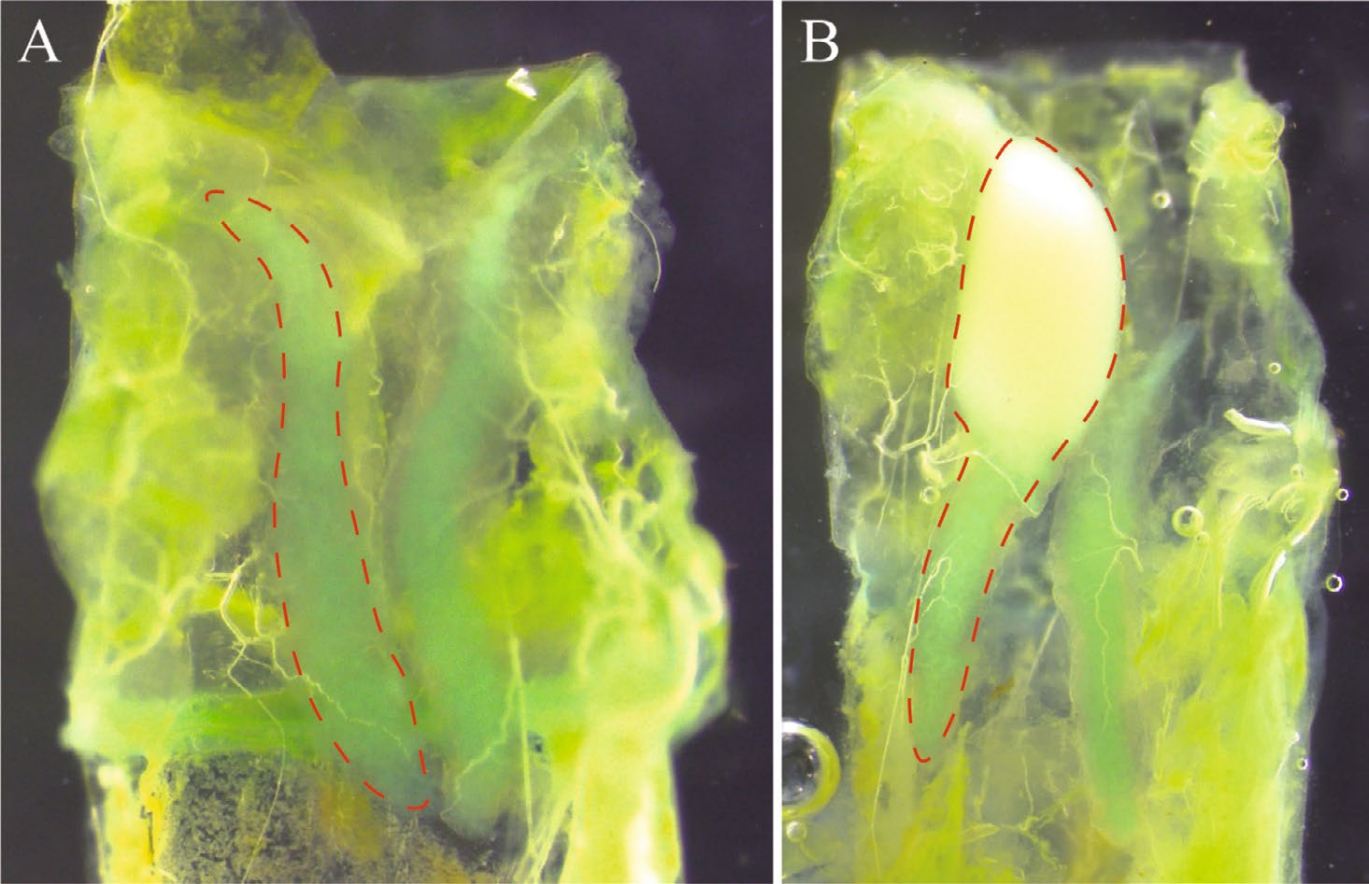
Ventral view of the prothoracic glands of *M. batesii* hatchlings, with the right gland outlined (photos: ACOV). (A) Gland containing little defence fluid. (B) Gland containing a substantial amount of defence fluid. The yellow‐white fluid inside the gland is similar in appearance and odour to fluid produced by *M. batesii* adults.

**FIGURE 3:**
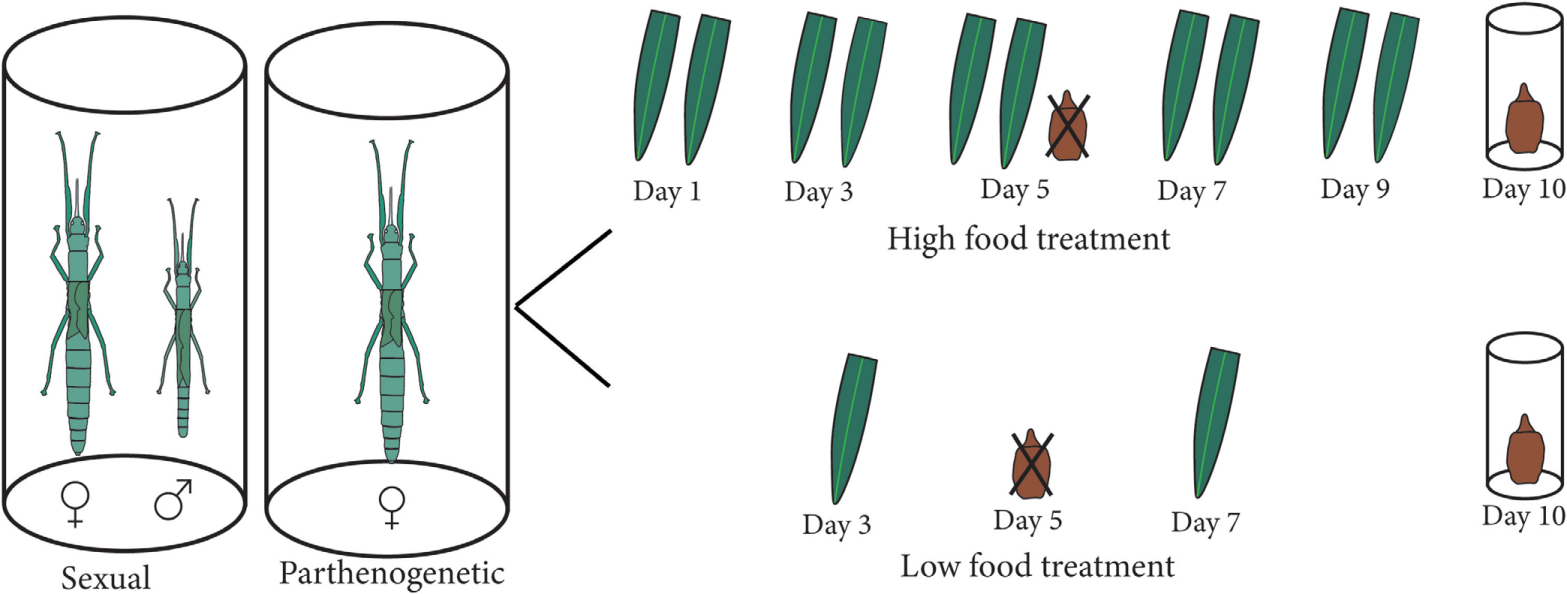
Experiment design: Male‐paired females (mostly sexual reproduction) and unpaired females (exclusively parthenogenetic reproduction) were allocated to either high food treatment or low food treatment for 10 days. The eggs laid over the first 5 days were discarded, and the eggs laid from the fifth to the tenth day were collected and placed into individual vials until hatching. After 10 days, each female or pair was switched to the other diet treatment: Adults that were in the high food treatment were switched to the low food treatment and vice versa for a further 10 days, and eggs were collected as described above (not shown). Thus, each individual female or pair experienced both diet treatments sequentially (low‐high or high‐low).

We discovered that some lab‐reared *M. batesii* were able to spray from their prothoracic ducts in response to handling immediately after hatching and prior to their first meal. This spray had a similar yellow‐white appearance to adult defensive spray and had the same peppermint‐like odour. This suggested that mothers could be provisioning their eggs with actinidine or its chemical precursor. We therefore investigated whether the defence fluid of hatchlings had the same chemical composition as the adult defence fluid and whether the same chemical compounds were also present in the eggs. We also observed that some hatchlings sprayed large amounts of defence fluid when handled (Video 2), while others sprayed little or none (Video 3), and that hatchlings varied considerably in the amount of defence fluid present in their prothoracic glands (Figure 2). We investigated two potential sources of variation in maternal provisioning: environmental resources (nutrition) of mothers and maternal mode of reproduction (sexual vs. parthenogenetic). We manipulated nutrition by providing females with low or high quantities of *Pandanus* leaves (low food vs. high food). We also manipulated reproductive mode by housing females alone or with a male in a full factorial design (Figure 3). We then tested for effects of reproductive mode, nutrition, and their interaction on investment in reproduction and offspring by measuring female fecundity (number of eggs and eggs size) and offspring traits (development time, hatching success, hatchling size, and prothoracic gland size). We used prothoracic gland size as a proxy of the amount of defence fluid possessed by hatchlings and the quantity of defence chemicals transmitted by females to their offspring.

**VIDEO 2:** *Megacrania batesii* hatchling that sprays defence fluid when provoked. The hatchling was placed into a zip‐lock bag and gently prodded on the head with tweezers. The video was recorded at 500 frames per second. Video content can be viewed at https://onlinelibrary.wiley.com/doi/10.1002/ece3.71243

**VIDEO 3:** *Megacrania batesii* hatchling that does not spray defence fluid when provoked. The hatchling was placed into a zip‐lock bag and gently prodded on the head with tweezers. The video was recorded at 500 frames per second. Video content can be viewed at https://onlinelibrary.wiley.com/doi/10.1002/ece3.71243

If fathers contribute resources that females can invest in offspring, or if mating induces females to invest more of their own resources in offspring, then we would expect female fecundity and offspring performance to be higher when females reproduce sexually (i.e., produce offspring from fertilised eggs) than when females reproduce parthenogenetically (i.e., produce offspring from unfertilised eggs). Sexual reproduction could thus result in an increased size or number of eggs (fecundity), shorter development time, higher offspring viability (hatching success), bigger hatchling sizes, and more actinidine provisioning. Sexually produced offspring also exhibit higher levels of heterozygosity than do parthenogenetically produced offspring in *M. batesii* (Miller, Stuart, et al. 2024a), which could result in higher offspring growth rates (egg development time and hatchling size) or viability (Mallet and Chippindale 2011). However, elevated offspring heterozygosity could not explain effects of mating on traits that are determined prior to fertilisation, such as the number of eggs, egg size, or the amount of diet‐derived defence chemicals transferred to eggs. In addition, if female investment in offspring is determined by their nutritional environment, then we would expect female fecundity and offspring performance to be higher when unpaired females (parthenogenetic reproduction treatment) or pairs (sexual reproduction treatment) have access to abundant resources (high diet treatment) than when they are food‐limited (low diet treatment).

## 2 Materials and Methods

### 2.1 Animal Maintenance

Eggs of *M. batesii* were collected from mixed‐sex populations of the Northern genotype and all‐female populations from the Southern genotype between Cow Bay and Cape Tribulation, Queensland (Miller, Stuart, et al. 2024a), in February and March of 2020 and raised in the lab at UNSW Sydney. Eggs were maintained in 125 mL plastic containers with moist cocopeat in a room with a controlled temperature of 27°C and watered periodically to prevent desiccation. Hatchlings (F0) were housed in full‐sib same‐sex pairs in cylindric plastic cages (20 × 40 cm) and fed leaves of *Pandanus tectorius* until they became adults. The F1 descendants of those individuals were used in the experiment described below, including 20 females produced by Northern genotype mothers and 24 females produced by Southern genotype mothers. Reproductive mode was manipulated for F1 females descended from both Northern and Southern populations. Twenty days after the females started to lay eggs, 22 females (14 Northern genotype and 8 Southern genotype) were paired with males, with each female–male pair housed in a separate cage (sexual treatment), while 22 females (6 Northern genotype and 16 Southern genotype) were kept alone (parthenogenetic treatment). The reproductive treatments were set up in the most ecologically relevant way for *M. batesii* , since adult females in all‐female populations are rarely found in close proximity with other adult females, but many adult females in mixed‐sex populations are guarded continuously by males (Boldbaatar et al. 2024). Consistent with previous reproductive mode manipulations on this species (Wilner et al. 2025), females in the sexual treatment laid mostly fertilised eggs, as indicated by the near‐even sex ratio of their offspring (34 males, 48 females, 41% male offspring), whereas females in the parthenogenetic treatment laid exclusively unfertilised eggs and produced exclusively female offspring (66 females).

### 2.2 Chemical Analyses

To investigate whether mothers of *M. batesii* were transferring their defensive chemicals to offspring, we used gas‐chromatography mass spectrometry analyses (GC–MS) to compare the chemical composition of egg yolk and hatchlings' prothoracic glands with the defence fluid sprayed by adults. For this analysis, we analysed the chemical composition of yolk from 18 eggs collected from randomly selected focal females (4 eggs from high food/parthenogenetic; 3 eggs from low food/parthenogenetic; 5 eggs from high food/sexual; 6 eggs from low food/sexual), prothoracic glands from 20 hatchlings produced by non‐focal females fed ad libitum (11 hatchlings produced by unpaired females and 9 hatchlings produced by male‐paired females), and defence fluid from 2 non‐focal adults fed ad libitum (one female, one male). The eggs were washed with ethanol and had their shell removed to expose the yolk. We dissected the prothoracic glands of hatchlings by cutting the ventral upper portion of the prothorax and removing the glands with a pair of fine forceps. Defence fluid was collected from live adults by tapping the prothorax with an insertion glass vial until the adults sprayed their defence fluid inside the vial. Yolk, prothoracic glands, and defence fluid were collected in insertion glass vials (300 μL). The amounts of yolk, prothoracic glands, and defence fluid were not consistent among samples, precluding quantitative comparisons of chemical concentrations. Extractions were performed on ice to avoid loss of volatile compounds. Each sample was transferred to a 1.5 mL glass vial, and 220 μL of chloroform was added and allowed to evaporate overnight at room temperature, leaving a small quantity of precipitate on the bottom of the vial. We then added 100 μL of acetonitrile to each sample and vortexed each vial. The vials with the extracts were placed immediately into a GC–MS instrument for the analyses of the chemical composition of the samples.

Chemical analyses of all extracts were carried out on a Focus DSQ GC–MS equipped with a Triplus autosampler (Thermo Fisher Scientific, Germany). Separations were carried out using a HP‐5MS capillary column with 30 m × 0.25 mm of internal diameter and 0.25 mm of film thickness (19091S‐433, J&W Scientific, USA). A spitless injection with a 4 μL injection volume was used, with the injector temperature set to 230°C. The source temperature was set at 200°C, and the start time (solvent delay) was set at 5 min. Oven temperature was as follows: initial temperature 60°C (held for 2 min), then from 60°C to 115°C (held for 2 min) with a heating rate of 6.5°C/min, then from 115°C to 125°C (held for 1 min) with a heating rate of 1°C/min, and from 125°C to 325°C (held for 9 min) with a heating rate of 7.2°C/min. GC–MS data were processed on the qual browser in Xcalibur software (version 2.1, Thermo Scientific), and spectra matched against the Wiley NIST library (NIST wiley 9 NIST 11) using the NIST Search 2.0 programme for identifications.

### 2.3 Diet Manipulation

We implemented two feeding treatments (high food and low food) and two reproduction treatments (sexual and parthenogenetic) in a full factorial design (Figure 3). In order to maximise statistical power to detect effects of the diet manipulation, we used a within‐subjects design where each female or pair experienced both feeding treatments sequentially, with each treatment lasting 10 days. Because female or pair identity was included as a random effect in the analysis, we were thus able to account for individual variation by comparing the performance of each individual female on the high versus low diet. Half of the experimental adults from each reproduction treatment were assigned initially to the high food treatment and half to the low food treatment (first diet treatment). After the first diet treatment was completed, adults that were in the high food treatment were switched to the low food treatment and vice versa (second diet treatment). In the high food treatment, female–male pairs or single females received two pieces of leaf (~10 cm in length) every second day (five feedings over 10 days) (Figure 3). At each feeding, the sizes of remaining leaves from the previous feeding were recorded and the old leaves were then discarded. The high food treatment can be considered as *ad libitum* feeding (75% of the total leaf area was eaten). Leaves were eaten mostly by the females as adult males eat much less than females (Boldbaatar 2022). In the low food treatment, female–male pairs or single females received one piece of leaf (~10 cm in length) on the third and seventh days of treatment (two feedings over 10 days) (Figure 3). The size of the leaf was recorded, and the leaf was discarded after 2 days from the day of feeding. Insects in the low food treatment ate 88% of total leaf area, with the unconsumed part of the leaf often dry or covered by the clip used to hang the leaf. Individual plants could vary in nutritional quality and availability of chemical precursors for actinidine synthesis. However, because leaves from food plants were provided randomly to females across different treatment combinations, such variation is unlikely to bias our results. We also assume that *Megacrania batesii* regulate their feeding and avoid over‐feeding. These animals nearly always have access to abundant food (host plant leaves) in the wild (RB, personal observations), and it is thus unlikely that they would over‐feed if provided with excess leaves in the lab.

Prior to the experiment, several females were dissected to estimate the length of time required for eggs to develop fully in the ovaries. We observed that eggs in different stages of development, including five to six eggs almost fully yolked, were present in the females' ovaries and oviduct. Therefore, we opted for discarding the eggs laid over the first 5 days after the start of the feeding manipulation. Females of *M. batesii* lay approximately one egg per day, and those first five laid eggs would have been fully or almost fully yolked prior to the diet manipulation and therefore unlikely to be affected by the treatments. Eggs laid in the second half of the treatment (last 5 days) were collected, photographed, and placed into separate containers with a layer of moist cocopeat at the bottom. The eggs were separated into individual containers so that hatchlings could be frozen without provoking the defence spray behaviour. We recorded the number of eggs laid, eggs size, egg development time (days from laying to hatching), and egg hatching success (proportion of eggs that hatched). The eggs, hatchling thorax, and hatchlings' dissected prothoracic glands were photographed using a camera (Leica MC170HD) mounted on a stereoscope (Leica MZ16A, Wetzlar, Germany) with lighting standardised by using a consistent light source and blocking out ambient light. Traits were then measured from the images using ImageJ (Schneider et al. 2012). We measured the length and width of the eggs (mm), the length of the thorax (cm), and the area of the prothoracic glands (calculated as the product of length and maximum width in pixels) as a proxy for the amount of defence fluid present in the glands.

### 2.4 Statistical Analyses

Feeding treatment (high vs. low food), reproductive mode (pairing with a male vs. no pairing), maternal genotype (Northern vs. Southern genetic cluster), order of feeding treatment application (low → high vs. high → low), and all interactions among diet, reproduction, and genotype were modelled as fixed effects using the R package glmmTMB (Brooks et al. 2017). Gaussian error distribution was used to model egg development time, egg size (arbitrary units), hatchling size, and prothoracic gland size (log‐transformed, arbitrary units); Poisson error distribution was used to model the number of eggs laid, and binomial error distribution was used to model egg hatching success (hatched vs. unhatched outcome for each egg). Mother identity was modelled as a random effect in all models to account for repeated measures on individual focal females, but paternal identity was not modelled because parthenogenetically produced females had no father. We modelled prothoracic gland size with hatchling body size (thorax length) included as an additional fixed covariate to account for variation in body size among hatchlings. Because female and male hatchlings are very similar in body size and hatchling sex did not explain variation in any measures of hatchling performance, we did not include hatchling sex in the models.

Akaike's Information Criterion corrected for small samples (AIC_c_) was used to determine support for covariates and interactions by comparing models containing all possible subsets of the fixed effects from the above glmmTMB models (but retaining maternal identity as a random effect in all models) using the “dredge” function in the MuMIn package (Bartoń 2024). The null (intercept‐only) model was included among the models compared for each response variable. The top model and other models with ΔAIC_c_ < 2 were considered to have statistical support (Burnham and Anderson 2002; Burnham et al. 2011; Johnson and Omland 2004; Symonds and Moussalli 2011), and model‐averaged effect coefficients were calculated based on all supported models using the “model.avg” function. As a measure of model fit, the marginal *R* ^2^ (proportion of total variance explained by the fixed effects) and conditional *R* ^2^ (proportion of total variance explained by both fixed and random effects) were calculated for the full model using the “r.squaredGLMM” function. All statistical analyses were conducted using R version 4.1.0 (R Core Team 2021).

## 3 Results

### 3.1 Chemical Analysis

**FIGURE 4:**
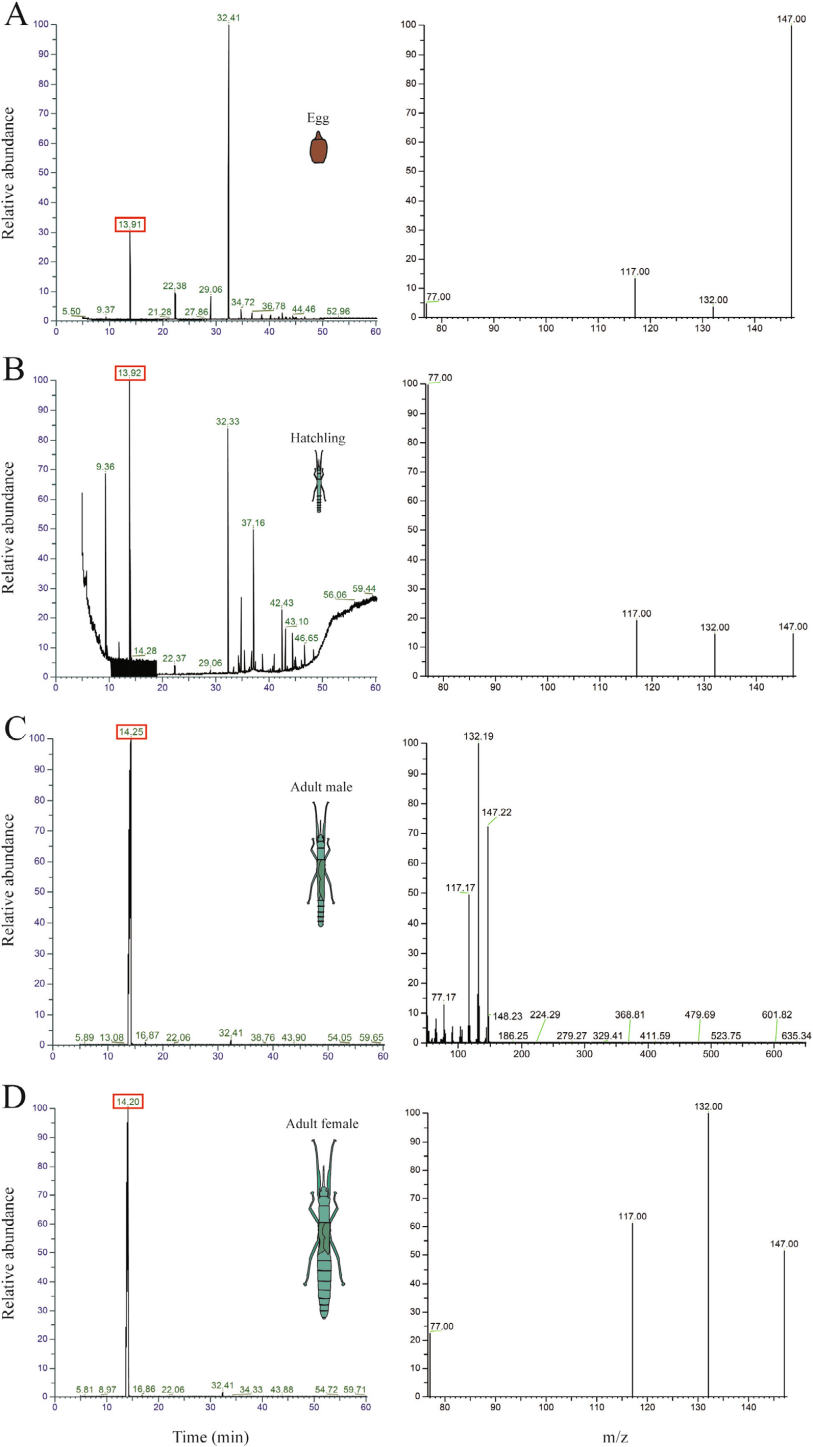
GC–MS analysis of representative samples. Relative abundance refers to the MS signal strength (arbitrary units). of a total ion chromatogram. The retention time of the actinidine peak is highlighted in red in the left panels. The mass spectral data (mass to charge ratio, m/z) of the actinidine peak are shown on the right. Spectra of the yolk of an egg (A). Spectra of the prothoracic glands of a hatchling (B). Spectra of the spray of an adult male (C). Spectra of the spray of an adult female (D).

Comparison of GC–MS chromatograms from the different extractions revealed the presence of a compound containing similar masses in all sample types: this compound was observed at similar retention times in the egg yolk (retention time = 13.91) and hatchling gland (retention time = 13.92), and both the adult male defence fluid (retention = 14.25) and adult female defence fluid (retention time = 14.20) (Figure 4). The peak containing the compound had the same masses (77 m/z, 117 m/z, 132 m/z, 147 m/z) in egg, hatchling, and female samples (Figure 4A,B,D). In the male, the compound masses were slightly bigger (77.17 m/z, 117.17 m/z, 132.19 m/z, 147.22 m/z), but this difference was well within the range of measurement error (0.4 Da) for the GC–MS instrument (Figure 4C). We compared the mass and retention time of this compound to chemicals in the Wiley NIST (w9n11) library of the NIST MS software and found that this compound closely matched the mass spectrum of the alkaloid actinidine.

### 3.2 Fecundity and Egg Size

**FIGURE 5:**
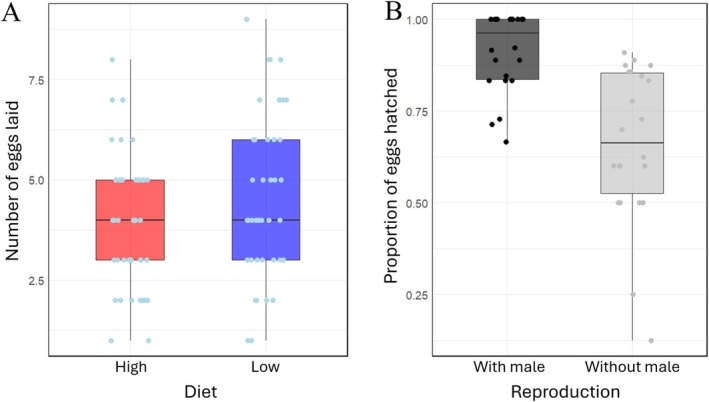
(A) Number of eggs laid over the last 5 days of the 10‐day diet treatment by focal females on high (red) and low (blue) diets. Each focal female was exposed sequentially to both diet treatments, such that each female's fecundity is represented by a point in each diet treatment group. (B) Proportion of eggs that hatched, shown for focal females that were paired with males and therefore laid at least some fertilised eggs (black) and females that reproduced without males and therefore laid unfertilized eggs that developed parthenogenetically (grey). Each point represents the proportion of hatched eggs from a single focal female. Boxes show inter‐quartile range, bars show medians, and whiskers span the 5%–95% percentile range.

For the number of eggs laid over the 5‐day egg collection period, two of the four supported models included an effect of diet: females from the low food treatment laid ~15% more eggs than did females from the high food treatment (Figure 5A). Two of the supported models also included an effect of order, with females laying more eggs during the first diet treatment than during the second diet treatment. However, the null model was also among the top models, and both marginal and conditional *R* ^2^ were low (Table 1).

**TABLE 1:** Model comparisons based on AIC_c_ for the egg number (number of eggs laid by focal females during the last 5 days of diet treatment application), egg size (egg length × egg width), development time (number of days from oviposition to hatching), hatching success (hatched or unhatched outcome for each egg), and hatchling body size (thorax length).

Intercept	Order	Genotype	Reproductive mode (R)	Diet	GxD	GxR	DxR	GxTxR	df	LogLik	AIC_c_	ΔAIC_c_	Weight
Egg number ($R_{\text{marginal}}^{2}$ = 0.07, $R_{\text{conditional}}^{2}$ = 0.07)													
+	+								3	−177.12	360.53	0.00	0.14
+	+			+					4	−176.12	360.73	0.20	0.12
+									2	−178.32	360.78	0.25	0.12
+				+					3	−177.42	361.12	0.60	0.10
1.461	−0.163			0.143									
Egg size ($R_{\text{marginal}}^{2}$ = 0.11, $R_{\text{conditional}}^{2}$ = 0.61)													
+	+		+						5	−386.46	783.08	0.00	0.20
+	+		+	+					6	−385.61	783.44	0.36	0.17
+	+		+	+			+		7	−385.08	784.46	1.39	0.10
+	+								4	−388.40	784.90	1.83	0.08
+	+	+	+						6	−386.41	785.06	1.98	0.08
8.276	−0.431	0.068	0.448	−0.059			−0.134						
Development time ($R_{\text{marginal}}^{2}$ = 0.30, $R_{\text{conditional}}^{2}$ = 0.49)													
+	+		+						5	−474.42	959.26	0.00	0.21
+	+		+	+					6	−473.47	959.54	0.28	0.19
+	+		+	+			+		7	−472.64	960.08	0.82	0.14
+	+	+	+						6	−474.23	961.06	1.79	0.09
116.47	−4.589	−0.871	−5.809	−0.668			−2.312						
Hatching success ($R_{\text{marginal}}^{2}$ = 0.21, $R_{\text{conditional}}^{2}$ = 0.28)													
+			+						3	−167.49	341.05	0.00	0.27
+	+		+						4	−167.36	342.83	1.78	0.11
+		+	+						4	−167.47	343.04	2.00	0.10
0.799	−0.146	−0.077	1.705										
Hatchling body size ($R_{\text{marginal}}^{2}$ = 0.07, $R_{\text{conditional}}^{2}$ = 0.21)													
+	+	+		+					6	293.15	−573.70	0.00	0.08
+		+							4	290.93	−573.57	0.13	0.07
+		+		+					5	291.99	−573.57	0.14	0.07
+		+		+	+				6	293.07	−573.54	0.17	0.07
+	+	+							5	291.83	−573.23	0.47	0.06
+	+			+					5	291.80	−573.17	0.53	0.06
+				+					4	290.56	−572.85	0.86	0.05
+									3	289.41	−572.66	1.05	0.05
+	+	+		+	+				7	293.72	−572.63	1.07	0.05
+	+								4	290.38	−572.47	1.23	0.04
+	+	+	+	+					7	293.51	−572.22	1.48	0.04
+		+	+						5	291.31	−572.19	1.51	0.04
+		+	+	+					6	292.35	−572.11	1.59	0.04
+		+	+	+	+				7	293.34	−571.87	1.83	0.03
+	+	+	+						6	292.21	−571.82	1.88	0.03
0.886	−0.008	0.014	0.006	0.010	−0.014								

*Note:* All models included fixed effects of order of High versus Low diet treatment application (“Order”), focal female's descent from the Northern versus Southern genetic cluster (“Genotype”), focal female's pairing versus non‐pairing with a male (“Reproductive mode”), focal female's High versus Low diet treatment (“Diet”), and all interactions among these factors. Focal female identity was included as a random effect in all models. For each response variable, the top model (ΔAIC_c_ = 0) and other supported models (ΔAIC_c_ < 2) are listed, with model degrees of freedom (“df”), log‐likelihood (“LogLik”), AIC_c_, ΔAIC_c_, and weight shown. Factors included in the supported models are indicated by “+” and model‐averaged coefficients are shown below the list of supported models. Marginal andconditional *R* ^2^ for the full model are shown for each response variable.

**FIGURE 6:**
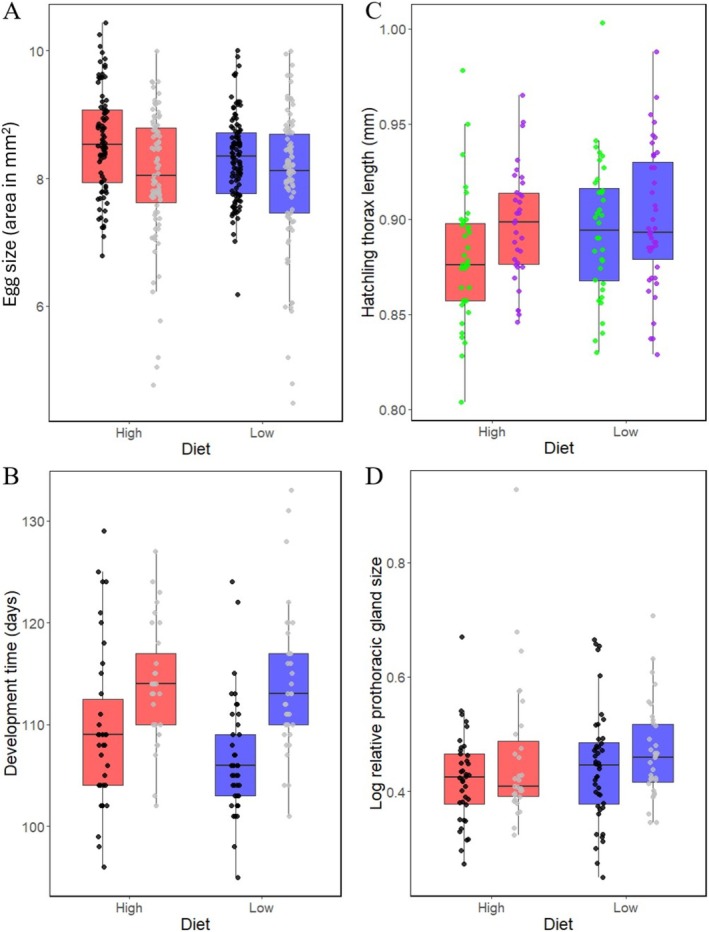
(A) Sizes of eggs (egg area in mm^2^) laid by females on high (red) and low (blue) diets and reproducing with males (black points) or without males (grey points), with each point representing a single egg. (B) Development time (days from oviposition to hatching) for eggs laid by females on high (red) and low (blue) diets and reproducing with males (black points) or without males (grey points), with each point representing a hatched egg. (C) Hatchling body size (thorax length in cm) of offspring of females maintained on high (red) and low (blue) diets and descended from the northern genetic cluster (green points) or southern genetic cluster (purple points), with each point representing a hatchling. (D) Relative prothoracic gland area (ratio of log‐transformed combined area of the right and left glands to thorax length) of hatchlings produced by females maintained on high (red) and low (blue) diets and reproducing with males (black points) or without males (grey points), with each point representing a hatchling. Boxes show inter‐quartile ranges, bars show medians, and whiskers span 5%–95% percentile ranges.

For egg size, most of the supported models included reproduction, with females that were paired with males laying eggs that were ~5% larger than those laid by females that were housed without males (Figure 6A; Table 1). Two of the supported models (with ΔAIC_c_ = 0.36 and 1.39) also included diet treatment, with high food females laying eggs that were ~1.6% larger on average than those laid by low food females, and one model (ΔAIC_c_ = 1.39) also included a diet × reproduction interaction. All supported models also included order, with larger eggs laid during the first diet treatment than during the second diet treatment. One model included genotype, but support for this model was less strong (ΔAIC_c_ = 1.98).

### 3.3 Offspring Traits

For egg development time, all supported models included an effect of reproduction, with eggs laid by male‐paired females (i.e., mostly fertilised eggs) developing ~5% faster than eggs laid by unpaired females (i.e., unfertilised eggs) (Figure 6B; Table 1). In addition, two supported models (ΔAIC_c_ = 0.28 and 0.82) included an effect of diet and one model included a diet × reproduction interaction, with male‐paired females on a high diet producing eggs that developed ~4% faster than male‐paired females on a low diet. All supported models also included an effect of order, with eggs laid during the second diet treatment developing faster than eggs laid during the first diet treatment. One model included an effect of genotype, but support for this model was less strong (ΔAIC_c_ = 1.79).

For hatchling success, all supported models included an effect of reproduction, with eggs laid by male‐paired females (i.e., mostly fertilised eggs) having a ~38% higher probability of hatching than eggs laid by unpaired females (i.e., unfertilised eggs) (Figure 5B; Table 1). One model also included an effect of order, and one model included an effect of genotype, but support for these models was less strong (ΔAIC_c_ > 1.78).

For hatchling body size, many models had some level of support, including the null model (ΔAIC_c_ = 1.05). Nearly all supported models included an effect of genotype, with Southern genotype hatchlings being ~1.5% longer on average than Northern genotype hatchlings (Figure 6C; Table 1). Most supported models also included an effect of diet, and three models included a diet × genotype interaction, with Northern genotype hatchlings being ~1.7% shorter when produced by high diet females than when produced by low diet females. Finally, some models included an effect of order, with hatchlings produced during the second diet treatment being smaller than hatchlings produced during the first diet treatment.

For prothoracic gland size, all supported models included an effect of body size (thorax length), indicating that larger hatchlings had larger prothoracic glands. The best‐supported model included an effect of reproduction: the prothoracic glands of hatchlings produced by unpaired females (i.e., from unfertilised eggs) were ~8% larger than those of hatchlings produced by male‐paired females (i.e., from mostly fertilised eggs) (Figure 6D; Table 2). Although one supported model lacked an effect of reproduction, this model had weaker support than the best model (ΔAIC_c_ = 1.38). One supported model included effects of both reproduction and diet, but this model was also less strongly supported (ΔAIC_c_ = 1.49).

**TABLE 2:** Model comparisons based on AIC_c_ for the combined area of the prothoracic glands of hatchlings.

Int.	Body size	Order	Genotype (G)	Reproductive mode (R)	Diet	GxD	GxR	DxR	GxTxR	df	LogLik	AIC_c_	ΔAIC_c_	Weight
Prothoracic gland size ($R_{\text{marginal}}^{2}$ = 0.13, $R_{\text{conditional}}^{2}$ = 0.14)														
+	+			+						5	480.53	−950.63	0.00	0.20
+	+									4	478.77	−949.26	1.38	0.10
+	+			+	+					6	480.87	−949.14	1.49	0.09
0.458	0.0898			0.0030	0.0013									

*Note:* All models included fixed effects of hatchling body size (thorax length), order of High versus Low diet treatment application (“Order”), focal female's descent from the northern versus southern genetic cluster (“Genotype”), focal female's pairing versus non‐pairing with a male (“Reproductive mode”), focal female's High versus Low diet treatment (“Diet”), and all interactions among these factors. Focal female identity was included as a random effect in all models. The top model (ΔAIC_c_ = 0) and other supported models (ΔAIC_c_ < 2) are listed, with model degrees of freedom (“df”), log‐likelihood (“LogLik”), AIC_c_, ΔAIC_c_, and weight shown. Factors included in the supported models are indicated by “+” and model‐averaged coefficients are shown below the list of supported models. Marginal and conditional *R* ^2^ for the full model are also shown.

## 4 Discussion

We found that *M. batesii* eggs and hatchlings are provisioned with the defence chemical actinidine. Moreover, we found that the size of the prothoracic glands of hatchlings, where the defence fluid is stored, was influenced by reproductive mode, with hatchlings produced from unfertilised eggs having larger glands than hatchlings produced from fertilised eggs. Reproductive mode also affected egg size, development time, and hatching success. Maternal diet affected egg size and development time, and interacted with genotype to affect hatchling body size. Overall, our results suggest that sexual reproduction increased some aspects of female allocation to eggs and enhanced egg viability, whereas parthenogenetic reproduction appeared to enhance offspring provisioning with defence chemicals. Our findings thus reveal that *M. batesii* eggs and hatchlings are subject to a complex combination of maternal effects associated with both maternal nutrition and reproductive mode.

*Megacrania batesii* nymphs and adults spray copious amounts of defence fluid in response to simulated predator attack (Jones and Bulbert 2020). As in other species of *Megacrania* (Chow and Lin 1986; Ho and Chow 1993; Prescott et al. 2009), the active ingredient in this defensive spray—the alkaloid actinidine, which causes severe irritation if sprayed into the eyes of vertebrate predators—appears to be synthesised from diet‐derived chemical precursors. The ability to spray defence chemicals immediately after hatching might be highly advantageous as well. *M. batesii* females drop their eggs on the ground or into crevices between leaves (Cermak and Hasenpusch 2000). After hatching on the ground and prior to their first meal, hatchlings must disperse to host plants—a phase when they are highly vulnerable to predation by ants, spiders, or small vertebrates. Having the ability to spray during this phase could therefore increase survival to the adult stage. It is not known how long it takes for *M. batesii* hatchlings to sequester and accumulate actinidine through feeding. Thus, maternal allocation of actinidine to hatchlings might continue to be advantageous even after the hatchlings find a host plant and begin to feed. Eggs might also benefit from containing actinidine if actinidine deters predators from eating the eggs. Moreover, actinidine is known to have antifungal properties (Saxena and Mathela 1996) and could thus reduce the rate of fungal infections in eggs and hatchlings. Particularly high rates of infection occur in all‐female populations of *M. batesii* that inhabit swamp areas (Miller, Stuart, et al. 2024a; Miller, Wilner, et al. 2024b). Thus, hatchlings from these populations might benefit considerably if actinidine in their tissues and/or spray can prevent fungal growth.

The effects of maternal diet that we detected were relatively subtle and sometimes unexpected. For example, we found some evidence that low diet females laid more eggs, although statistical support for this effect was tentative. We also found that eggs laid by high diet females took longer to develop. We found no effect of diet on egg hatching success, no overall effect on hatchling body size, and weak evidence that high maternal diet was associated with larger prothoracic glands in hatchlings. The lack of clear evidence of positive effects of high maternal diet might be due to females being able to store fat in their abdomens and use those fat reserves when there is no food available in the environment (Arrese and Soulages 2010). If so, it is possible that our 10‐day diet manipulations were not long enough to induce clear effects of diet. Longer‐term variation in food abundance during the adult stage, or variation in resource availability throughout development, could affect reproductive effort and offspring provisioning to a greater extent or in a different way. For example, females that develop on a high diet might be larger as adults and lay more or larger eggs (e.g., Steiger 2013). Alternatively, it is possible that well‐fed mothers allocated fewer resources per egg and instead produced more eggs as a strategy that enhances maternal fitness at the expense of individual offspring fitness (Uller 2008), as observed in some species (e.g., Einum and Fleming 2000). Indeed, oviposition rate is likely to trade off against egg quality (Hammers et al. 2013; Lemaître et al. 2015; Smith and Fretwell 1974). Although the low food females were not starved completely, it is also possible that the severe reduction in food abundance in the low food treatment triggered females to elevate their oviposition rate because they perceived an elevated risk of death from starvation. Likewise, food limitation could have affected male sperm quality or allocation, and competition with males for food could have resulted in more severe food limitation for paired females than for single females. However, we found little support for diet × reproductive mode interactions, suggesting that any such effects were minor.

Mating enhanced several offspring traits. This might have occurred because sexually produced offspring are more heterozygous, because males transfer resources to females at mating, or because males induce females to invest more of the females' own resources in offspring. Our findings that sexual reproduction had a positive effect on egg development time, offspring viability, and hatchling size could be explained by higher heterozygosity of sexually produced offspring (see Miller, Stuart, et al. 2024a). Evidence of positive heterozygosity effects on offspring viability and fitness, based on the comparison of inbred and outbred individuals, has been reported in many species (e.g., Pekkala et al. 2014; Taylor et al. 2010). Our findings that sexually produced eggs had higher viability are also consistent with some findings of other studies on facultatively parthenogenetic systems (e.g., Engelstädter 2008; Kobayashi and Miyaguni 2016), including stick insects (Burke and Bonduriansky 2018, 2022; Wilner et al. 2025). Our results are also consistent with paternal provisioning of resources to females at mating (e.g., Garcia‐Gonzalez and Simmons 2010), or stimulatory effects of chemicals in the seminal fluid, such as seminal fluid proteins (Avila et al. 2011; Simmons 2011; Sirot et al. 2015; Wagner and Harper 2003; Wigby et al. 2016). In particular, given that eggs are fully provisioned prior to fertilisation, such stimulatory effects could explain why male‐paired females laid larger eggs.

We found that parthenogenetically produced hatchlings had larger prothoracic glands than did sexually produced hatchlings, suggesting that maternal provisioning of offspring with defence chemicals was negatively affected by sexual reproduction. Although we did not quantify actinidine content in hatchlings produced by focal females from our experimental manipulation of maternal reproduction and diet, we found that hatchlings contain actinidine and store defence fluid in their prothoracic glands similarly to adults. It is therefore likely that the enlarged prothoracic glands of parthenogenetically produced hatchlings contained greater amounts of actinidine, although further work is required to determine whether maternal diet or reproductive mode affects the concentration of actinidine in the defence fluid of hatchlings. While there is evidence of paternal provisioning of offspring with defence chemicals in some insects (e.g., Eisner and Meinwald 1995), our findings suggest that *M. batesii* males do not transfer actinidine to females at mating. Instead, our results suggest that actinidine provisioning is enhanced by a maternal effect associated with reproduction without males. This maternal effect could result from the costs imposed by guarding males on females. Sexually reproducing *M. batesii* females carry guarding males almost continuously (Boldbaatar et al. 2024). Interaction with males could therefore involve considerable energetic costs for females (Watson et al. 1998) and perhaps impose increased risk of predation and infection, potentially resulting in sexual conflict in the wild (Wilner et al. 2025). Females that reproduce without males could therefore have more resources to invest in the chemical defences that they transmit to their offspring. We also found tentative evidence of effects of maternal diet on hatchlings' prothoracic gland size, but it is possible that longer‐term or early‐life variation in food availability could have stronger effects. In addition, it is possible that females regulate actinidine allocation to their eggs based on perceived risk of predator attack or fungal infection—a possibility that requires further investigation.

We found that several traits were affected by the order of diet treatment application, with fewer and smaller eggs laid during the second diet treatment, and some evidence of reduced hatching success and hatchling body size. Conversely, eggs laid during the second diet treatment developed faster. The effect of treatment order could result from female ageing during the experiment. However, this explanation seems unlikely because *M. batesii* adults can live for 1 year in laboratory conditions (R.B., unpublished data). The reasons for this effect thus require further investigation.

In summary, we found evidence that eggs are provisioned with the defence chemical actinidine, enabling hatchlings to spray defence fluid prior to their first feeding. Moreover, our experimental results suggest that a maternal effect associated with parthenogenetic reproduction enhances the amount of defence fluid in hatchlings' prothoracic glands. Both maternal diet and reproductive mode also affected other reproductive and offspring traits. Further studies that experimentally manipulate reproductive mode in facultatively parthenogenetic systems are required to clarify how mating and environmental conditions influence female fitness, parental transmission of resources to offspring, and offspring fitness.

## Author Contributions

**Ana Caroline Oliveira Vasconcelos:** conceptualization (equal), data curation (lead), formal analysis (lead), investigation (lead), visualization (equal), writing – original draft (lead), writing – review and editing (equal). **Lewis Adler:** data curation (supporting), formal analysis (supporting), methodology (supporting), supervision (supporting). **Russell Bonduriansky:** conceptualization (equal), data curation (supporting), formal analysis (supporting), funding acquisition (equal), methodology (supporting), project administration (equal), resources (equal), supervision (lead), visualization (supporting), writing – review and editing (equal).

## Conflicts of Interest

The authors declare no conflicts of interest.

## Data Availability

Data and code are available from the Dryad Digital Repository: http://datadryad.org/stash/share/jFAs5uX7XeNHSyB_5ppuqqQJc3e5bXsOEcfSJvHCrG8.
